# Mineral Plastics and Gels from Multi‐Arm Ionomers

**DOI:** 10.1002/gch2.202400244

**Published:** 2025-01-12

**Authors:** Neta Shimony, Adi Gross, Boaz Mizrahi

**Affiliations:** ^1^ Faculty of Biotechnology and Food Engineering Technion – Israel Institute of Technology, Technion City Haifa 3200003 Israel

**Keywords:** biomaterials, degradation, mineral plastics, multi‐arm, poly(acrylic acid)

## Abstract

Plastic production and waste are a growing menace that affects the soil, the marine environment, and the air in a cumulative manner. The demand for mineral and bioplastics from renewable and biodegradable materials has therefore increased in all relevant sectors. The use of currently available degradable plastics is, however, limited by their poor mechanical properties and high production costs. In addition, many of today's plastics undergo uncontrolled biodegradation processes that involve harsh or expensive conditions and which may last from months to years. Here, the advantages of using multi‐arm polymers for the production of sustainable mineral plastics are presented. A 4‐arm poly(acrylic acid) is synthesized via atom transfer radical polymerization and is reacted with divalent calcium ions to obtain semi‐liquid hydrogel or degradable plastic when dried. The mechanical properties of the different phases are evaluated and compared with linear poly(acrylic acid) of the same molecular weight. The multi‐arm approach yielded improved mechanical characteristics, including self‐healing and biodegradation without compromising other typical hydrogel characteristics. This concept of synthesizing multi‐arm polymers with improved characteristics from building blocks of traditionally linear structures may be applicable to other mineral and bioplastic materials including acrylates, polysaccharides, and DNA.

## Graphical abstract

The characteristics of four‐arm poly acrylic acid are compared with those of linear polymer as elastic hydrogels and mineral plastics. While in the hydrogel state, poly acrylic acid backbone has no significant effect on the mechanical properties, when dried, the four‐arm plastics presented superior mechanical, self‐healing, and degradation performances, which provide an elegant strategy for designing next‐generation mineral plastics from known materials.

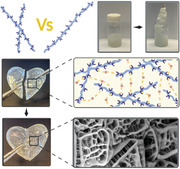

## 1 Introduction

The environmental outcomes of fossil fuel extraction and of plastic accumulation have motivated the development of sustainable, biodegradable materials.^[^ ^1^ ^]^ The demand for biodegradable plastic from renewable materials has recently increased significantly in all sectors.^[^ ^2^ ^]^ In the materials science community, sustainable plastics have been the focus of widespread interest mostly owing to their hierarchical organization, micro‐ and nanoscale order, high surface area, and lightweight properties. As a result of these efforts, biodegradable materials, together with green technologies, have recently yielded novel sustainable materials including liquids, plastics, fibers, and gels with a wide selection of properties and conformations.^[^ ^3^ ^]^ Inspired by living organisms that exploit minerals, especially divalent and trivalent cations (e.g., Ca^2+^, Mg^2+^, and Fe^3+^) for green crosslinking, a variety of inorganic‐organic hybrid materials can be designed for various functions.^[^ ^4^ ^]^ From a structural perspective, the crosslinking can be viewed as junctions bonding, where the strands are typically linear polymer chains including polysaccharides, glycoproteins, and recently segments of DNA.^[^ ^5^ ^]^ Once the 3D network is formed between the positive divalent cation and the negatively charge polymer, ionotropic hydrogels exhibit plastic‐like mechanical properties that, when dry, offer superior performance in terms of stiffness, strength, and flexibility. Since the supramolecular structure is based on non‐covalent electrostatic interaction, the final plastic can degrade quickly in the presence of chelating agents (e.g., citrate, EDTA, phosphate), high concentrations of competing ions (e.g., Na^+^ or K^+^), or in acidic solutions.^[^ ^6^ ^]^ Finally, unlike typical thermoplastics whose reshaping or recycling requires heat, mineral plastics can not only be processed under ambient temperatures but also result in more complex and precisely controlled architectures due to the plasticizing effect of surrounding water.^[^ ^7^ ^]^ Despite the recognized potential of degradable plastics, their widespread adoption for commercial use is restricted by their limitations.^[^ ^8^ ^]^ Mineral and other degradable plastics, in general, cost at least twice as much as conventional plastics, in particular when their building blocks require sophisticated.^[^ ^9^ ^]^ Another shortcoming of current degradable plastics is the complex nature of their biodegradation processes. Bacterial biomass, multiple bioenzymes, or mixing with biofuel byproducts are often needed in order to reach full degradation, which further increases the cost of production.^[^ ^10^ ^]^ Finally, these biodegradation methods may take months to years before full degradation is achieved. Here, we studied the effect of polymer architecture on the physical and mechanical properties of mineral plastic. While linear polymers such as poly(acrylic acid) (PAA) have received significant attention as acrylic plastic components, the development of multi‐armed (star‐shaped) polymers remains a relatively unexplored topic. In the present research, we hypothesized that 4‐arm PAA may overcome some of the inherent deficiencies of linear PAA systems owing to the unique properties of multi‐arm backbones: a more compact structure with higher elasticity and lower viscosity compared with linear polymers of the same molecular weight.^[^ ^11^ ^]^ The lower intrinsic viscosity of multi‐arm polymers has been attributed to the sharp decrease in dynamic entanglement as the number of arms increases.^[^ ^12^ ^]^ In addition, compared with their linear counterparts, multi‐arm structures lead to more intra‐molecular and intra‐chain hydrogen bonds and to a lower proportion of hydrogen bonds with surrounding water molecules.^[^ ^13^ ^]^ In this study, we synthesized 4‐arm PAA with a molecular weight of 117 000 Da using atom transfer radical polymerization (ATRP). We chose this molecular weight since it was found to be optimal for plastic formation.^[^ ^14^ ^]^ Then, we compared the relevant properties of 4‐arm PAA with those of linear PAA of similar molecular weight. The effect of the PAA structure, both as a hydrogel and a dry plastic material, on various parameters was studied.

## 2 Results and Discussion

**Figure 1:**
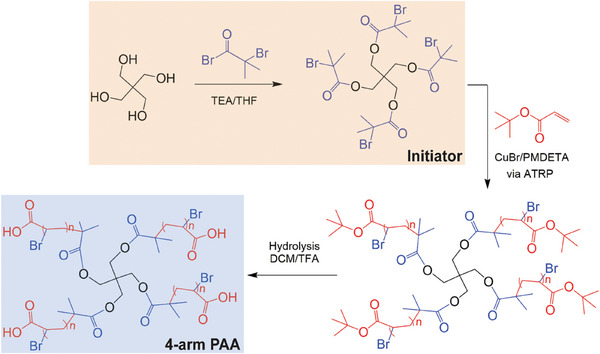
Synthesis scheme of 4‐arm PAA via ATRP followed by hydrolysis. First, the initiator 4‐arm pentaerythritol tetrakis(2‐bromoisobutyrate) was synthesized using BIBB as the initiator. Then, tert‐butyl acrylate was polymerized via ATRP followed by hydrolysis to obtain the final product, 4‐arm PAA.

The initiator 4‐arm pentaerythritol tetrakis(2‐bromoisobutyrate) was synthesized by reacting pentaerythritol with α‐bromoisobutyryl bromide (BIBB) in the presence of triethylamine (TEA, **Figure** **1**). ^1^H‐NMR confirmed 98% substitution of BIBB (Figure S1, Supporting Information). Fourier transform‐infrared (FTIR) spectrometer (ALPHA, Bruker, MA, USA) revealed a new peak at 2970 cm^−1^ that we attributed to C─H bonds formed simultaneously with the disappearance of O─H bonds at 3300 cm^−1^ (Figure S2, Supporting Information).

Synthesis of 4‐arm PAA was carried out by a two‐step reaction (Figure 1): First, the synthesized 4‐arm pentaerythritol tetrakis(2‐bromoisobutyrate) (0.25 g, 0.00034 mole) was reacted with tert‐butyl acrylate (t‐BA) (61.5 g, 0.48 mole) via ATRP followed by hydrolyses with dichloromethane (DCM) and trifluoroacetic Acid (TFA) to remove the tert‐butyl groups. ^1^H‐NMR confirmed the successful synthesis and purity of the final 4‐arm PAA by the presence of peak at 1.5 ppm during the synthesis (Figure S3, Supporting Information) and the complete removal of tert‐butyl groups (Figure S4, Supporting Information); This result was also confirmed by FTIR (Figure S2, Supporting Information). Size exclusion chromatography with multi‐angle laser light scattering (SEC‐MALLS‐RI, Figure S5, Supporting Information) plots of 4‐arm PAA indicated a molecular weight of 117kDA, implying good agreement with the theoretical value.

**Figure 2:**
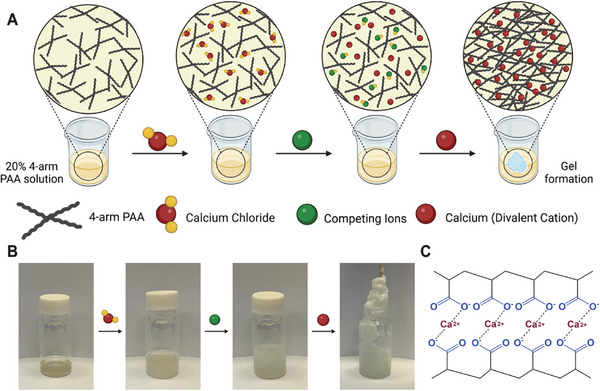
Formation of PAA gels. A) Schematic illustration of the creation of electrostatic cross‐linked gel. B) Photographs of gel formation over time. C) Chemical structure of cross‐linked PAA gel showing electrostatic bonds between PAA chains and calcium ions.

The PAA/Ca^2+^ gel samples were synthesized by dropwise addition of either 4‐arm or linear PAA solutions (20% W/V) into saturated CaCl_2_ solution, followed by dropwise addition of Na_2_CO_3_ 2 m solution while stirring. This synthetic scheme, inspired by biomineralization processes, allows the formation of amorphous calcium carbonate (ACC) nanocrystals that spontaneously form nanosegregated structures when physically crosslinked with PAA^[^ ^15^, ^19^ ^]^ (**Figure** **2**A). Indeed, within a few seconds, a sticky white precipitate began to form on the magnetic stirrer and on the vessel walls (Figure 2B). After ≈1 min, both linear and 4‐arm PAA solutions were discarded, and the sticky martial was washed with deionized water several times until the washing solutions were clear and stretchable, soft yet tough gels were formed. The fast gel formation is attributed to the strong electrostatic interaction between Ca^2+^ ions of the ACC and the PAA's numerous carboxyl groups^[^ ^16^ ^]^ (Figure 2C).

**Figure 3:**
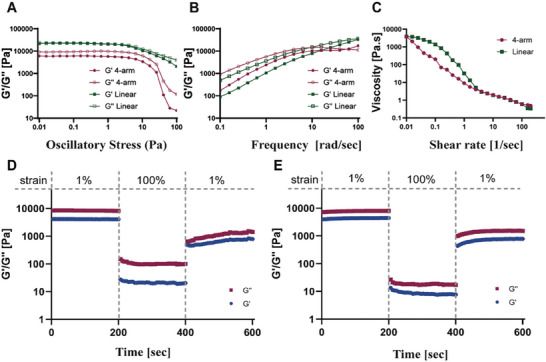
Rheological behavior of the PAA/Ca^2+^ hydrogel. A) Linear viscoelastic region (LVR) B) Frequency dependencies of the storage (G’) and loss (G”) moduli of the two hydrogels. C) Viscosity as a function of shear rate of the two hydrogels. D) 3ITT recovery test of 4‐arm PAA. E) 3ITT recovery test of linear PAA.

The rheological behavior of the 4‐arm PAA/Ca^2+^ and of the linear PAA/Ca^2+^ hydrogels were assessed using a rheometer. We first determined the linear viscoelastic region (LVR) of the hydrogels (**Figure** **3**A). Within the LVR, the *G*″ values were equal or greater than the *G*′ values, indicating that the PAA solutions presented similar or higher viscous character compared to the elastic one. The viscoelastic moduli of the linear and of the 4‐arm PAA hydrogels presented an initial plateau‐like region followed by a decrease in a stress range of 5–10 Pa. Based on this observation, the strain amplitude of 1% was determined to keep the deformation in the LVR. In both 4‐arm and linear polymers, the loss modulus was slightly higher than the storage modulus (G″ > G′) over the entire frequency range (Figure 3B), implying the formation of weak precipitates. Moreover, In both polymers, storage, and loss moduli were found to increase with an increase in frequency, which can be explained by the partial crosslinked network structure of PAA.^[^ ^17^ ^]^ Storage modulus (G′) and loss modulus (G″) values were close and in the range between 0.1 and 50 rad s^−1^. The difference between the storage and the loss moduli started decreasing above 50 rad s^−1^, until emergence was observed ≈90–100 rad s^−1^. The close modulus values are indicative of the semi‐liquid state of both the 4‐arm and the linear PAA hydrogels, as was documented for other PAA‐based hydrogels as well.^[^ ^18^ ^]^ It is noteworthy that the higher storage modulus and loss modulus (102–105), relative to commonly used hydrogels, is a strong indication of the strong mechanical properties of the PAA hydrogels, which can be explained by the large amounts of calcium ions incorporated in them.^[^ ^19^ ^]^ Viscosity was tested using the flow‐sweep test at a shear rate of 0.01–100 1/s. The viscosity of the hydrogels was reduced significantly (<1 Pa.s) as the shear rate increased to 100 1/s (Figure 3C). Thus, it can be concluded that the polymers’ structure had a negligible effect on materials’ rheological behavior in the hydrogel state.

The effect of polymer structure on the recovery rate of the hydrogel was investigated using a three‐interval thixotropy test (3ITT). Tests were performed at 1% strain (within the LVR), then at 100% strain (outside of the LVR), and again at 1% strain (Figure 3D,E). Cycles were conducted for 200 s at room temperature. In both hydrogels, viscosity values dropped by two orders of magnitude at 100% stain (high shear) and recovered by one order of magnitude immediately after the strain was lowered back to 1% (low shear). Both hydrogels maintained liquid state with loss moduli values constantly above their storage moduli values (G″>G′) throughout the entire test. Interestingly, at 100% strain, G′ and G″ values of the 4‐arm hydrogel were ≈four times higher than those of the linear hydrogel (110 and 40 Pa vs 30 and 10 Pa, correspondingly), manifesting that the 4‐arm hydrogel is more pronounced to influence on its shear thinning behavior. Nonetheless, the 4‐arm system demonstrated a slightly better recovery rates compared to the linear system (20% vs 16%, respectively), as was evident by the moduli values when the strain was reduced back to 1%. These observations can be explained, at least partially, by the initial arrangement and more ordered structure of the 4‐arm gel, resulting in a higher amount of polymer chain interaction via electrostatic bonds.^[^ ^20^ ^]^ In addition, the more elastic structure of the 4‐arm gel may also contribute to the recovery rate by allowing the chain extra flexibility thus contributing to faster recreation of the electrostatic bond between PAA and calcium ions.

**Figure 4:**
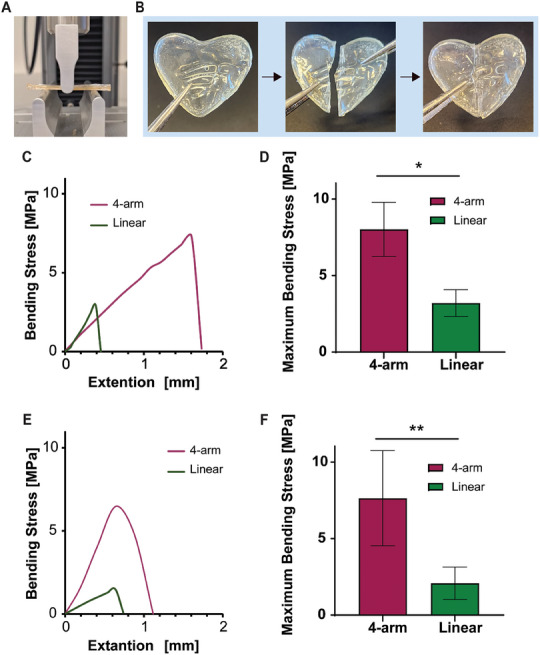
Mechanical and self‐healing properties of PAA mineral plastics. A) Photograph of the three‐point bending apparatus. B) Self‐healing demonstration, the broken plastic recovers in a wet condition. C) Representative force‐extension curve of 4‐arm PAA and linear PAA plastics. D) Plastics breaking force *n* = 5, * = *p* < 0.0001. E) Representative force‐extension curve of 4‐arm PAA and linear PAA plastics after self‐healing. F) Plastics breaking force after self‐healing *n* = 5, ** = *p* < 0.01.

Self‐healing, the capability to heal spontaneously after breakage, is an important characteristic of crosslinked plastics containing electrostatic COO^−^/Ca^2+^ bonds with dynamic exchange characteristics are expected to exhibit self‐healing capabilities.^[^ ^21^ ^]^ The mechanical properties of the plastics, as well as their self‐healing capabilities, were quantitatively assessed by the three‐point bending flexural test until failure (**Figure** **4**A). The 4‐arm plastic exhibited self‐healing capabilities (Figure 4B) an elastic region that corresponds to a linear profile, followed by a more plastic region and a continuous decrease in the stress‐strain slope. The plastic broke down at ≈8MPa (Figure 4C,D). The linear system exhibited only a plastic region, which broke down at 3MPa.

To assess the self‐healing capabilities, after each test, the two parts of the broken specimen were re‐attached by adding a little water to the top surface of the plastic and holding the two interfaces in light contact to achieve healing. After air‐drying for 2 days, the healed 45 × 10 mm samples were tested again using the same method (Figure 4E,F). While the 4‐arm plastic exhibited close to full recovery in terms of breaking stress (95%), the linear system achieved only 60% recovery, under the same conditions.

The fact that only the 4‐arm PAA plastic demonstrated healing properties can be explained by the high chain segment density at the surface of multi‐arm polymers, compared with their linear analog.^[^ ^22^ ^]^ Similar results have been observed for other linear polymers and their star‐like counterparts. For example, the self‐healing ability of linear and multi‐arm‐shaped copolymers from a naturally derived maltotriose core with poly(n‐butyl acrylate) and poly(tert‐butyl acrylate) (PtBA) segments was tested.^[^ ^23^ ^]^ Here too, only the star‐shaped copolymers exhibited full self‐repair/self‐healing of the damage after 30 min without external stimuli supporting the process. The improved self‐healing of the multi‐arm structure was explained by the higher number of individual subunits taking part in the self‐healing process.

**Figure 5:**
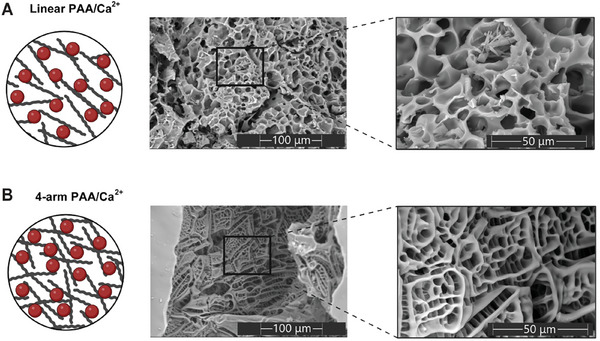
Illustration and SEM imaging of the freeze‐dried plastics A) Linear PAA/Ca^2+^. B) 4‐arm PAA/Ca^2+^.

The enhanced self‐healing performance of the 4‐arm plastic was possibly due to its more compact structure, and so, we next used SEM to study the structure of linear and 4‐arm PAA‐plastics (**Figure** **5**A,B). Linear PAA showed a disordered 3D network structure with interconnected pores ranging in size from 15 to 25 µm. In comparison, the 4‐arm PAA gel exhibited a compact structure, with round to quadrate pores containing many parallel structures surrounded by dense 5–20 µm thick walls. The more ordered, square‐shaped pore architecture of the 4‐arm PAA‐plastics can be attributed to polymer star structure, which enhances cross‐linking density, thus facilitating the formation of a more interconnected network due to an increase in physical entanglements and hydrogen bond interactions between polymer chains.^[^ ^24^ ^]^ Similar pore morphology has been found for other crosslinked linear PAA and star polymers hydrogels.^[^ ^25^ ^]^ For example, an 8‐arm star poly (ethylene glycol) crosslinked with a collagen‐based peptide was also found to consist of 5–25 µm round to square compartments.^[^ ^26^ ^]^ Pore size of the 4‐arm structure was in the range of 5–10 µm and this relatively small pore size can be explained by the high crosslinking density of the 4‐arm hydrogel.^[^ ^27^ ^]^ These results confirm the effect of PAA morphology on the structure and validate the quality of our explanation regarding the superior mechanical properties and self‐healing of the 4‐arm structure.

**Figure 6:**
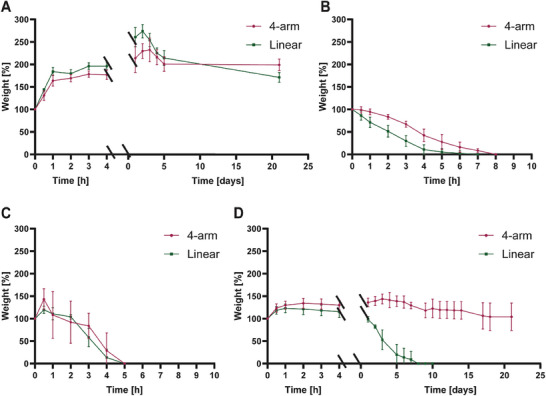
Degradation behavior of 4‐arm and linear PAA/Ca^2+^ plastics in various solvents at room temperature. A) In PBS. B) In EDTA. C) At pH 10. D. At pH 2. The results are reported as means ± SD (*n* = 3).

The degradation behavior of the PAA plastics was investigated with respect to the swelling ratio in PBS (pH 7.4) and EDTA solution, and under different pH conditions (**Figure** **6**). Linear and 4‐arm PAA plastics incubated in PBS swelled ≈100% and 50% in the first hour, respectively, following by slower swelling of an additional 100% and 150%, respectively, over the first 3 days. Limited degradation, amounting in ≈50% of their initial weight, was observed over the following 3 days, after which both polymers maintained their weight for 3 weeks. In EDTA solution, both polymers started degrading immediately upon submersion, without any noticeable swelling. The degradation time of the linear PAA was ≈5 h, while the 4‐arm PAA degraded completely after 8 h. At pH 10, both polymers showed a similar pattern, with full degradation after ≈5 h. Finally, at pH 2, both polymers swelled by ≈25% of their initial weight in the first 2 h and remained stable for ≈1 week. However, while the linear PAA completely degraded within 8 days, the 4‐arm PAA was not fully degraded even after 21 days.

Although some differences were observed, the swelling and degradation behavior of both linear and 4‐arm PAA in PBS, EDTA, and at pH 10 were similar. In PBS, the high degree of swelling is attributed to the absorbance of water by the electrostatic crosslinking bonds.^[^ ^28^ ^]^ We note that the swelling and degradation profiles of the linear system appear slightly pronounced compared with the 4‐arm system, possibly due to its amorphous nature that enables fairly rapid water uptake. This may also explain the faster degradation profile observed for the linear system in EDTA, as the chelating agent penetrates the structure quicker and compromises the electrostatic bonds between PAA and calcium ions, thus leading to faster swelling and degradation.^[^ ^29^ ^]^

Interestingly, at pH 10, both PAA plastics demonstrated comparable swelling and degradation profiles, with a pKa of ≈4.5, which is attributed to the multiple carboxylic acid groups on its chain.^[^ ^30^ ^]^ At pH 10, the carboxylic groups are protonated, resulting in an open network architecture caused by electrostatic repulsion between the COO^−^ groups.^[^ ^31^ ^]^ As a result, the structural differences between the 4‐arm and the linear PAAs are insignificant and negligible. This repulsion also contributes to the fast degradation of both PAAs, which takes place within only 5 h. In contrast, at pH 2, carboxylic groups are not protonated^[^ ^32^ ^]^ and continue to support the natural ordered structure of the two polymers. In addition, calcium ions are held relatively secure inside the crosslinked complex. Under these circumstances, the natively denser and more ordered 4‐arm structure leads to a more stable matrix. The degradation times of the 4‐arm PAA in the different media are a clear indication that a quick and controllable end‐of‐life degradation of this plastic can be obtained.

## 3 Conclusion

We successfully synthesized the initiator 4‐arm pentaerythritol tetrakis(2‐bromoisobutyrate) and used it to polymerize 4‐arm PAA using the ATRP technique. Fast gel formation was observed upon introduction of Ca^2+^ ions, owing to electrostatic interaction with carboxyl groups of the PAA. A mineral plastic can also be obtained by the removal of water. The effect of the 4‐arm structure, both in hydrogel form and as a dry plastic, was studied in comparison with linear PAA of similar molecular weight. In the hydrogel state, the PAA structure had no significant effect on the rheological and recovery properties; in the dry, plastic state, the 4‐arm system exhibited improved properties, including its mechanical, self‐healing, and degradation characteristics. It was also evident that PAA plastics can be degraded within several hours to a few days, in a controllable fashion and without the need of harsh chemicals, enzymes, or bacteria. The fact that the process of obtaining the plastic material is totally reversible upon immersing in water indicates good recyclability. These PAA plastics can be used in the food, pharmaceutical, and daily necessities industries where environmental friendliness, biocompatibility, self‐healing, and film‐forming products are desired. For example, PAA plastics can be used to replace conventional dry food and drug packaging, which are considered to be the main source of plastic waste pollution.^[^ ^33^ ^]^ These mineral plastics can also be used as edible films, fibers, and coatings as well as in the field of insulation applications, especially for heat insulation.^[^ ^34^ ^]^ Finally, PAA plastics have been found to be chemically and structurally stable, including after continuous exposure to neutral buffered (as was also demonstrated in our study), oxidizing, large‐molecule rich, and high‐temperature environments.^[^ ^35^ ^]^ Using the multi‐arm approach for the formation of novel mineral plastics may be applicable to other polymers including acrylates, polysaccharides, and DNA.

## 4 Experimental Section

### Materials

α‐Bromoisobutyryl bromide (BIBB), chloroform‐d (CDCl^3^), calcium chloride (CaCl_2_), copper bromide (CuBr), deuterium oxide (D_2_O), ethylenediaminetetraacetic acid (EDTA), magnesium sulfate (MgSO_4_), pentaerythritol (C(CH_2_OH)_4_), N,N′,N′′,N′′‐pentamethyldiethylenetriamine (PMDETA), linear poly(acrylic acid) (PAA), potassium chloride (KCl), triethylamine (TEA), tertbutyl acrylate (t‐BA), trifluoroacetic acid (TFA), sodium bicarbonate (NaHCO_3_), sodium carbonate (Na_2_CO_3_), and sodium hydroxide (NaOH) were purchased from Sigma–Aldrich (MO, USA). Hydrochloric acid (HCl), methanol (CH_3_OH), tetrahydrofuran (THF), dichloromethane (DCM), and diethyl ether ((C_2_H_5_)_2_O) were purchased from Bio‐Lab Ltd. (Jerusalem, Israel).

### Synthesis of 4‐Arm Initiator Pentaerythritol Tetrakis(2‐Bromoisobutyrate)

The 4‐arm initiator was synthesized following the procedures described by Xie et al.^[^ ^36^ ^]^ with a slight modification. In brief, 3 g pentaerythritol (136.15 g mol^−1^, 0.022 mol) was added to an ice‐cold round flask containing 20 mL TEA (101.191 g mol^−1^, 0.726 g mL^−1^, 0.144 mol) and 130 mL THF. A mixture of 20 mL BIBB (229.9 g mol^−1^, 1.86 g mL^−1^, 0.162 mol) in 5 mL THF was added dropwise to the pentaerythritol solution. The mixture was stirred at room temperature overnight, filtered, and concentrated by rotavapor to ≈10 mL. Then, 50 mL of DCM were added, and the solution was washed twice with saturated NaHCO3 solution, followed by one washing with HCl 1M, and a final washing with DDW. The organic solution was dried with anhydrous MgSO_4_, concentrated by rotavapor, and the final product was re‐crystallized from diethyl ether. ^1^H‐NMR and FTIR confirmed that 98% of the pentaerythritol was substituted by bromoisobutyrate.

### Synthesis of 4‐Arm Poly(acrylic acid)

Synthesis of the 4‐arm poly(acrylic acid) via ATRP was carried out under bulk conditions.^[^ ^37^ ^]^ In brief, t‐BA (61.5 g, 0.48 mol), CuBr (0.49 g, 0.0034 mol, 143.45 g mol^−1^), and PMDETA (0.72 mL, 0.0034 mol) were mixed in a Schlenk flask under N2 environment, after which 4‐arm initiator (0.25 g, 0.00034 mol, 732.09 g mol^−1^) was added. The mixture was deoxygenated by three freeze–pump–thaw cycles and heated in an oil bath to 80 °C still under N2. The reaction was stopped when the increased viscosity due to air exposure no longer enabled stirring. The mixture was diluted with THF after almost 2.5 h, and the polymer solution was passed through a natural alumina column to remove copper catalyst using THF as eluent. The eluted product was then concentrated by evaporation, precipitated into water/methanol (1:1), and dried under vacuum.^[^ ^38^ ^]^

The yellowish‐dried polymer was than hydrolyzed in a mixture of DCM and TFA. In brief, 1.5 g polymer was dissolved in 15 mL DCM. Then, 1.6 mL TFA was dissolved in 5 mL of DCM and the solution was added to the polymer mixture and left to hydrolyze at 40 °C for 48 h. The solvents were then removed by rotavapor and the polymer was washed with DCM and dried by exposure to air, yielding purified 4‐arm poly(acrylic acid).^[^ ^39^ ^]^

### Polymer Characterization

Chemical structures and substitution ratios of products from all reactions were determined in CDCl3 by 1H‐NMR, using a Bruker Avance III 400 MHz NMR spectrometer (Billerica, MA). Functionalization of copolymers was monitored using a Fourier transform‐infrared (FTIR) spectrometer (ALPHA, Bruker, MA, USA).

### Molecular Weight of 4‐Arm PAA Using SEC‐MALLS‐RI

A multi‐angle laser light scattering detector (MALLS) (PN3609) with RI Detector (PN3150), both from Postnova Analytics (Landsberg, Germany), were used after size exclusion separation at 35 °C using three columns (Waters, Milford, MA), namely, ultra‐hydrogel 250, 1000, and 2000 with exclusion limits of 8ˑ10^4^, 4ˑ10^6^, and 1ˑ10^7^ g mol^−1^, respectively. The sample was diluted with the eluent (0.1 m NaNO_3_) to a final concentration of 0.2% W/V and filtered (0.22 µm). The specific refractive index (dn/dc) of PAA was 0.1475 mL g^−1[^ ^40^ ^]^ the injection volume was 100 µL, and the flow rate was set at 0.5 mL min^−1^. Molecular weight was calculated by the random coil fitting method using the MALLS detector's software^[^ ^41^ ^]^ (Nova Mals, v. 1.5.0.7, Postnova Analytics, Germany).

### Gel and Plastic Preparation

PAA/Ca^2+^ gel samples were created by mixing a 20% W/V PAA solution (4‐arm or linear PAA) with saturated calcium chloride solution, followed by slow injection of 2M sodium carbonate solution into the stirred mixture. A white sticky precipitate formed gradually in a turbid solution containing non‐gelling fractions. The turbid solution was discarded, and the hydrogel was washed with deionized water several times until the washing solution was clear and an elastic gel was obtained. Plastics were parallelly obtained from the same hydrogels by air‐drying at room temperature for 2 days.

### Rheology

Rheological profiles of the synthesized polymers and the crosslinked hydrogels were evaluated on a Discovery Hybrid Rheometer (DHR‐2, TA Instruments, DE, USA) using parallel, 8‐mm‐diameter plates. The tested sample was placed between parallel plates at 25 °C and the gap between the plates was set to 1 mm. Excess sample was trimmed off the lower plate. The instrument was controlled using the Trios program (TA Instruments, DE, USA) and frequency‐sweep tests of each of the components were performed in the linear viscoelastic region (LVR) at a constant strain of 1%. The rheology behavior of each sample was characterized by its shear storage modulus (G′) and loss modulus (G″) at a constant strain of 1% in a frequency range of 0.1 rad s^−1^ to 100 rad s^−1^ using an oscillation‐sweep test. Viscosity was evaluated by a shear rate in the range of 0.01 1/s to 100 1/s.

Self‐recovery of the storage and loss moduli was evaluated by 3ITT, alternating the strain‐deformation measurements between 1% (in the LVR) to 100% (outside of the LVR) every 200 s.

### Three‐Point Bending Flexural Test

The fragility of the plastics was evaluated by the three‐point bending flexural test using a Lloyd TA1 texture analyzer (Lloyd Instruments Ltd., West Sussex, UK) equipped with a 50 N load cell for compression. Plastic samples (10 × 45 mm, 3 mm thick) were placed on two supporting pins set 21.5 mm apart. The third (loading) pin gradually applied load to the middle of the sample to detect the minimum stress [MPa] needed to break the samples. The results are reported as means ± SD (*n* = 5).

### Morphology

Plastics morphology was investigated using a wide‐field scanning electron microscope (the Netherlands; Model Quanta200 FEI ESEM), with a 20 kV accelerating voltage. The samples were frozen using liquid nitrogen, dried by lyophilization, and then coated by gold prior to imaging.

### Degradation

Samples (200 mg) of the PAA/Ca^2+^ gels (4‐arm or linear) were dried at room temperature for 3 days. The samples were then incubated at 25 °C in various buffers (pH 2, pH 7.4–PBS, pH 10, EDTA Solution) and shaken at 50 rpm until full degradation. The swollen samples were removed from the buffers at predetermined times, carefully blotted dry with filter paper, weighed, and returned to the incubator. The total percent of material weight was calculated by dividing the wet weight (Ww) of each swollen sample by its initial dry wet prior to incubation (Wd). The results are reported as means ± SD (*n* = 3).

### Statistical Analysis

Results of the three‐point bending flexural test are presented as mean values ± SD. Statistical comparisons (*p*‐value calculated) were performed with a Prism 10.2.3 GraphPad (La Jolla, CA, USA). T‐test analysis was used to test the significance of the differences between the two groups.

## Conflict of Interest

The authors declare no conflict of interest.

## Supporting information



Supporting Information

## Data Availability

The data that support the findings of this study are available in the supplementary material of this article.
