# Supplementary material for: Mineral Plastics and Gels from Multi‐Arm Ionomers
Source: Glob Chall. 2025 Jan 12;9(2):2400244. doi: 10.1002/gch2.202400244 (PMC11802327; doi:10.1002/gch2.202400244)
Supplement: Supplementary file 1 — Supporting Information [file GCH2-9-2400244-s001.docx]

# Supporting Information

# Mineral Plastics and Gels from Multiarmed Ionomers

Neta Shimony, Adi Gross, Boaz Mizrahi#

Faculty of Biotechnology and Food Engineering, Technion – Israel Institute of Technology, Technion City, Haifa 3200003, Israel.

^#^ Corresponding author, E-mail: bmizrahi@technion.ac.il

.


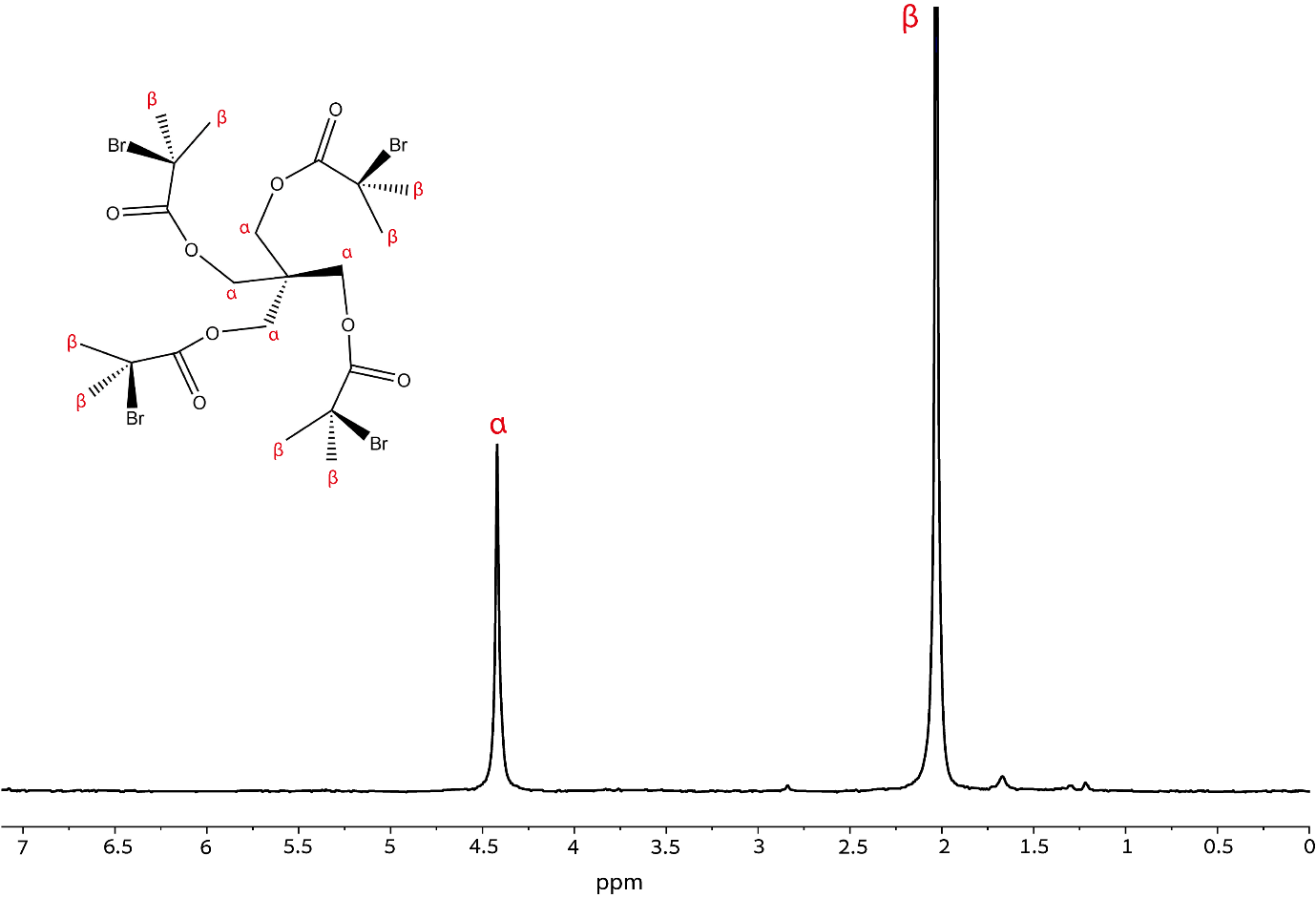


**Figure S1:** ^1^H NMR spectra of the initiator Pentaerythritol tetrakis(2-bromoisobutyrate). The presence of peaks around 2 ppm (marked by ß) confirmed 98% substitution.


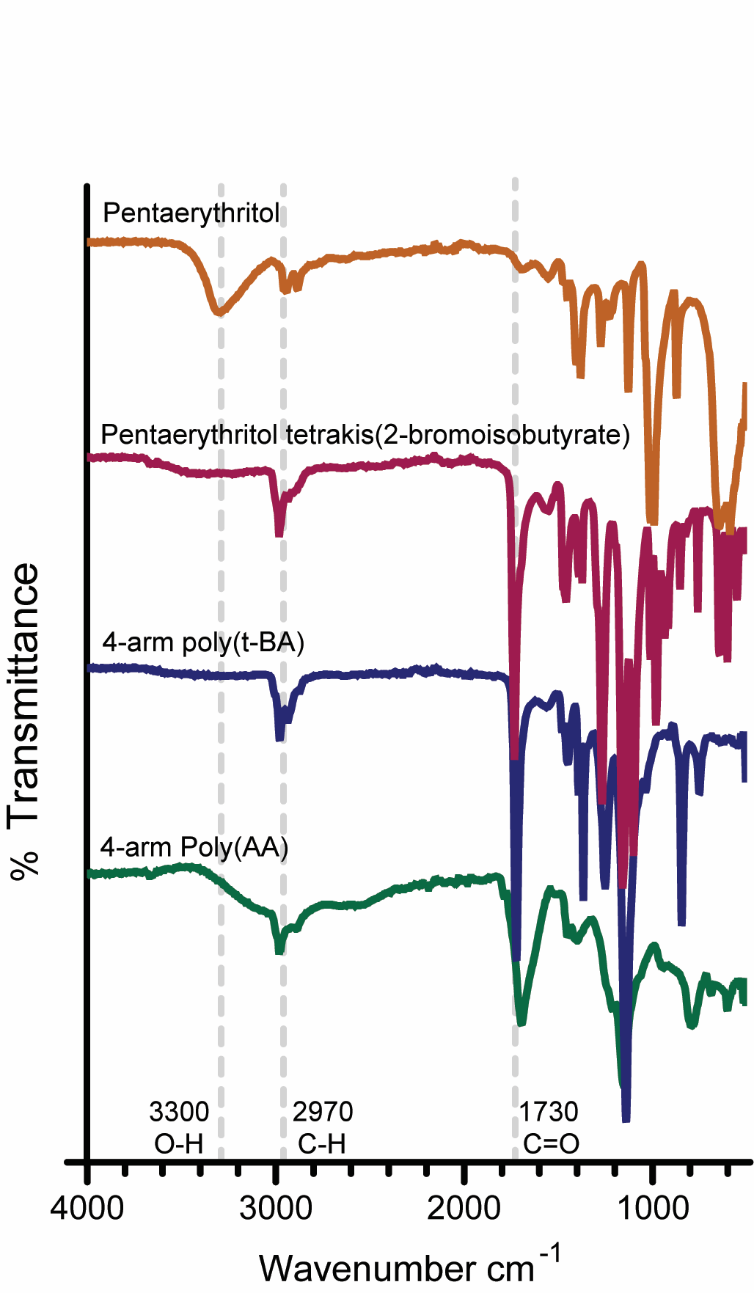


**Figure S2:** FTIR of Pentaerythritol, Pentaerythritol tetrakis(2-bromoisobutyrate) initiator, 4-arm Poly(t-BA) and 4-arm Poly(AA).

.
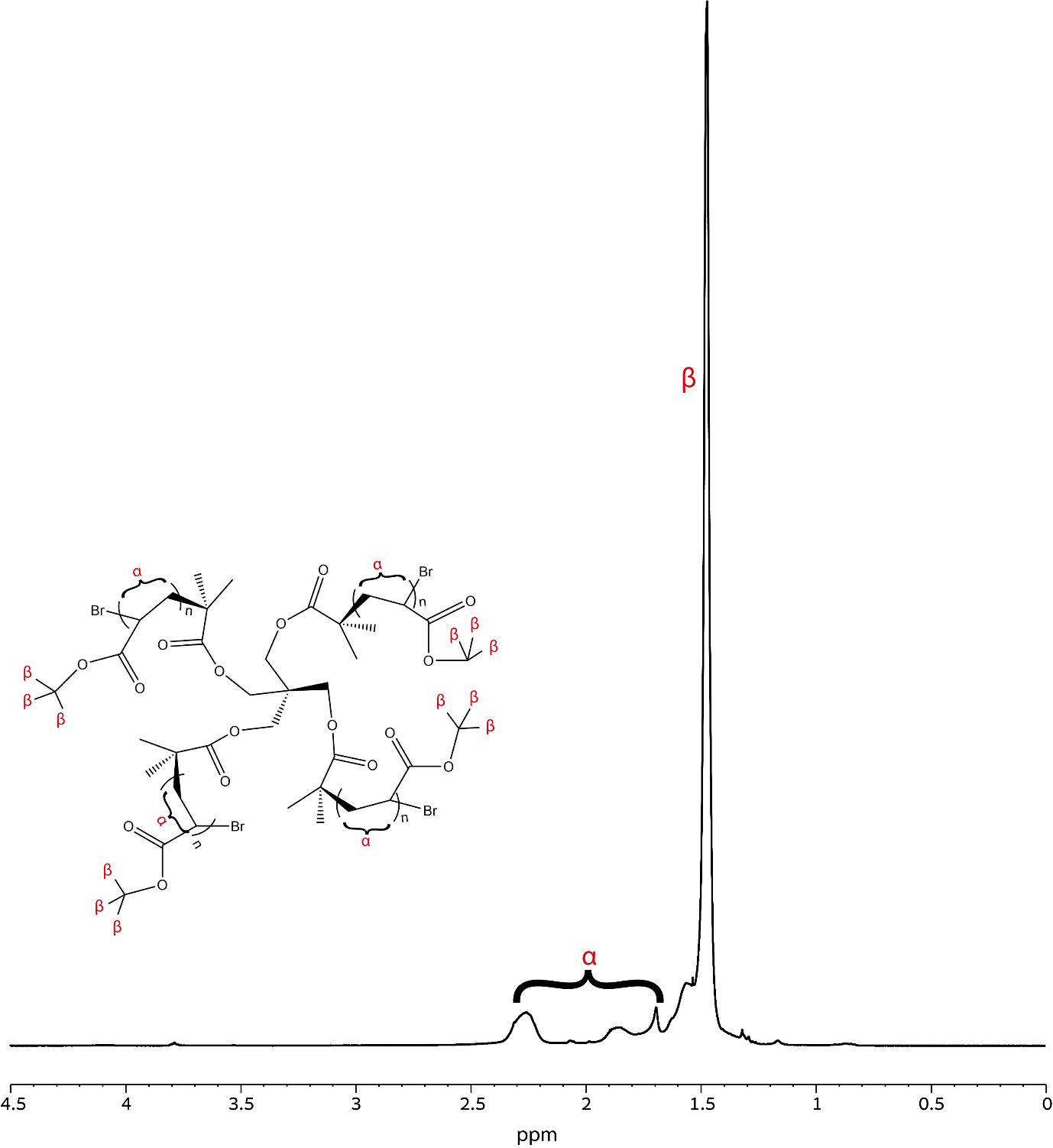


**Figure S3:** ^1^H NMR spectra of the synthesized PAA (before hydrolysis). The presence of the peaks at 1. 5 ppm (marked by β) confirmed the polymerization of t-Butyl Acrylate.


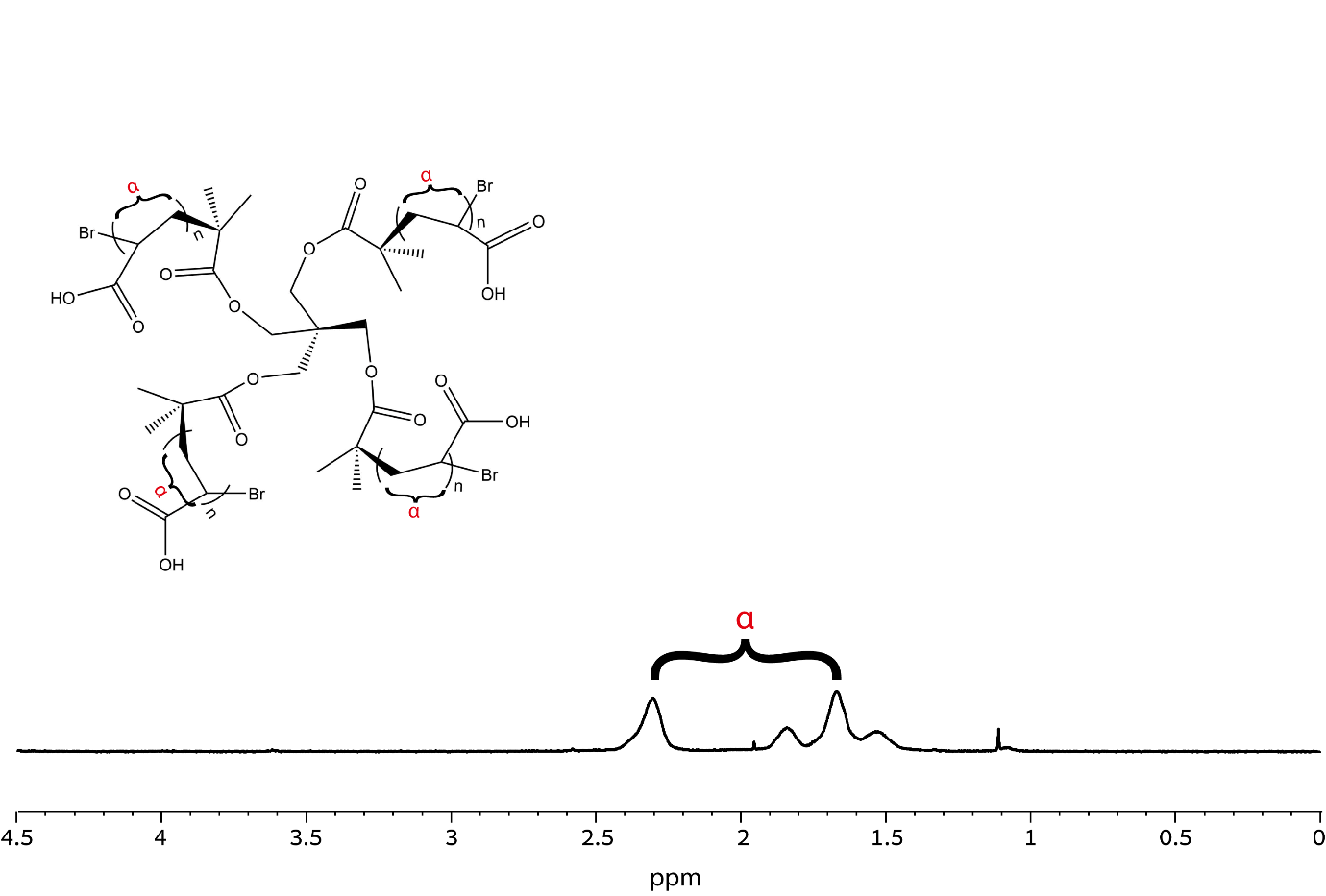


**Figure S4:** ^1^H NMR spectra of the synthesized PAA (after Hydrolysis). The absence of tert-Butyl peak (see in figure S3) confirmed the complete hydrolysis of tert-Butyl groups.


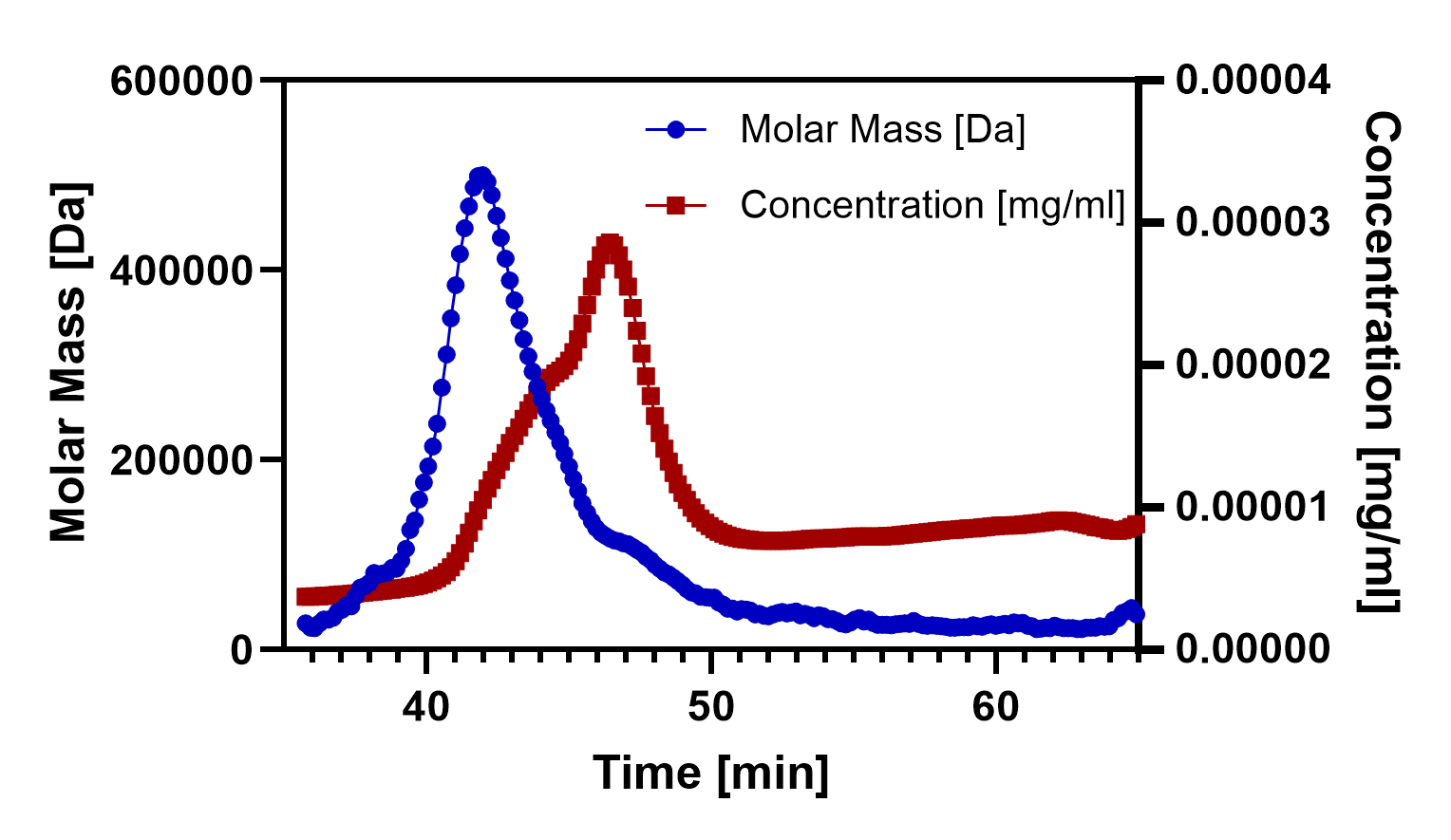


**Figure S5:** MALLS-SEC--RI plots of 4-arm PAA indicating molecular weight of 117kDA.
